# The Impact and Effectiveness of Virtual Reality Applied to the Safety Training of Workers in Open-Cast Mining

**DOI:** 10.3390/ijerph22020151

**Published:** 2025-01-23

**Authors:** Antonella Pireddu, Alessandro Innocenti, Luca Maurizio Lusuardi, Vincenzo Santalucia, Carla Simeoni

**Affiliations:** 1 Department of Technological Innovations and Safety of Plants, Products and Anthropic Settlements (DIT), National Institute for Insurance Against Accidents at Work (INAIL), 00144 Rome, Italy; 2 LabVR UNISI, Department of Social, Political and Cognitive Sciences (DISPOC), University of Siena, 53100 Siena, Italy; innocenti@unisi.it (A.I.);

**Keywords:** virtual reality, quarry, health and safety management, training

## Abstract

This paper presents the results of an interactive virtual reality (VR) training program aimed at enhancing Health and Safety (H&S) management practices in quarrying operations. The course was designed based on industry best practices, as well as both voluntary and mandatory standards relevant to marble mining activities. It combines experiential learning with a performance monitoring system that tracks completion rates, time taken, and scores based on user decisions. The primary objective was to assess the impact of VR training across different user groups, categorized by age, prior safety experience, familiarity with equipment and processes, and VR proficiency. This study involved 40 participants and analyzed 15 variables, including occupation, age, H&S skills, process knowledge, equipment familiarity, VR skills, physical impact of VR, number of attempts before completion, percentage and time of completion, achieved scores, retention of knowledge, and user feedback before and after training. Performance measurement was carried out using two methods: a Microsoft Forms questionnaire with 16 questions, completed by participants one week after training, and Simula Solution, which automatically tracked and recorded performance metrics (time, percentage, errors, and scores) during each session. The survey successfully identified which demographic groups were most affected by VR training. The findings of this study could have important implications for improving H&S practices in the mining sector by empowering workers to engage in training and interact with process resources. This allows them to experience virtual accidents in a controlled, risk-free environment.

## 1. Introduction

Occupational Health and Safety (H&S) training is a legal requirement in many countries across various sectors, highlighting its importance as a regulatory necessity [1]. In Italy, workers must complete Health and Safety courses in accordance with national legislation, which prescribes the frequency and methods of training. Traditionally, safety training has relied on videos, slides, and safety manuals, often fostering a passive learning environment [2,3]. Quarrying, however, can be dangerous, and ensuring safety remains a primary concern in this industry.

According to Innocenti [4], virtual reality is an effective method for simulating real-life situations and tasks, allowing precise control over the experiences encountered by users. Technically, VR refers to a computer-generated environment in which users interact in real time, creating artificial settings through interfaces that engage one or more senses. In VR, users’ movements are tracked, and the environment updates in sync with their actions.

VR technology can be applied in two types of environments, differentiated by the degree of user immersion. The first type is low-immersive virtual environments (LIVE), which are computer screen-based renderings of real or virtual worlds, such as Second Life, World of Warcraft, EverQuest, and The Sims. In these environments, users interact through digital avatars representing their virtual selves. The second type is high-immersive virtual environments (HIVE), which use specialized displays like the Cave Automatic Virtual Environment (CAVE), where images are projected onto multiple interior screens, or head-mounted displays such as Meta Quest, HTC Vive, or Pico. In HIVE environments, users’ senses are dominated by the equipment, with factors such as device adoption, field of view, rendering quality, and interaction speed influencing the experience.

As noted by Sabzevari and Dehghan [5], training plays a critical role in reducing workplace accidents and improving safety. Their 2018 study compared in-person and VR training on personal protective equipment (PPE) usage among 75 workers in an open stone quarry. Statistical analyses, including the Chi-square test, paired sample t-test, and ANOVA, showed no significant differences in PPE usage between the groups before training, but significant improvements were observed after the sessions.

Iran, which has one of the highest numbers of quarry mines globally, commonly uses diamond cutting wires in quarries to cut dimensional stone. However, these wires pose significant hazards. Identifying and addressing risks in quarries is crucial for ensuring safe and sustainable mining operations. In a study on quarry safety in West Azerbaijan, Esmailzadeh et al. applied the Failure Modes and Effects Analysis (FMEA) method to assess and rank hazards based on probability, intensity, and risk detection. The study identified diamond wire breakage, rock falls, and vehicle accidents as major risks in the region’s dimensional stone mines. The authors recommended preventive measures such as timely wire replacement, intelligent cutting tool control systems, comprehensive safety training, and adherence to safety protocols [6].

According to Nanadrekar et al., mining applications require specialized education for operators to enhance awareness and develop the necessary skills for their roles. The goal of training is to enable operators to perform their tasks in a manner that meets both company standards and safety requirements. The authors examined factors that contribute to the human efficiency of Dumper Operators in quarries, developing an Operator Skills Matrix (OSM) to guide retraining efforts when needed. Operators play a key role in improving safety and productivity, thereby reducing mining costs. Virtual Reality Training (VR) is one technique that can support this goal [7].

Renganayagalu et al. (2021) highlight the lack of review studies investigating the effectiveness of VR training. They argue for greater standardization of criteria used to assess the impact of VR as a training tool. This study aims to contribute to this effort by providing further insights into the effectiveness of VR in H&S training in the workplace [8].

The effectiveness of VR training varies by sector. In the healthcare industry, for example, VR has been widely used to train surgeons, physicians, and nurses. Many studies have validated the effectiveness of VR in surgical training, as it was one of the first areas to adopt VR technology. However, Stefan et al. found that in industries like construction, mining, agriculture, and transportation, VR training tends to be experimental and lacks efficacy assessments or widespread implementation [9].

Despite its potential, VR training has its limitations. Renganayagalu et al. examined professional training integrated with safety training, where safety was part of the broader production process. In such cases, assessing the effectiveness of VR for safety training in isolation becomes challenging [8]. Pedram et al. explored the technological aspects of VR and user experiences, emphasizing factors such as users’ attitudes toward technology, sector culture, and the adaptability of VR to specific training scenarios [10].

In Italy, national H&S training programs, as outlined in Legislative Decree No. 81 of 2008 and the State-Regions Agreements of 2011 and 2016, primarily involve classroom or e-learning sessions, with practical “in situ” tests required for emergency management and the use of hazardous machinery. However, general H&S training is not typically integrated into the production process, except where specifically mandated by national regulations.

This work is part of a project conducted under the research programs of the National Institute for Occupational Accident Insurance (Inail) and Siena University. It focuses on the evolution of smart safety technologies in the quarry sector. The project aims to develop a series of VR-based H&S training courses for workers and trainers in the open-pit quarrying industry. This study introduces the project, detailing its assumptions and focusing on the first of its four specific objectives (SO1). The goal of this objective is to evaluate volunteers’ ability to retain the VR training experience, identify errors made, and establish correlations with their previous skills. The hypothesis is that the simulation in SO1 adheres to Italy’s safety standards and best practices.

Additionally, a landscape component was introduced to replicate the altitudes of the Marble Quarries of the Apuan Alps, evoking the quarry’s isolation, slopes, and large marble blocks, which can reach 8–9 m in height. The simulation also incorporates sound effects such as wind at high altitudes and the noise of drilling and cutting machines. These elements aim to highlight the negative effects of noise on communication between workers, encouraging the use of gestures or other effective communication methods. A distractor scenario was included, in which an operator approaches the drilling area, risking injury. In this scenario, the user is required to stop the machine and move the operator to safety, emphasizing the importance of safety measures and the correct sequence of actions.

This adaptation of technology to the training scenario demonstrates the strength of VR in fostering greater awareness of risks. Innocenti suggests that VR experiments offer practical advantages, as researchers can conduct experiments in virtual environments tailored to specific needs. Costs are no longer a barrier to integrating such technology, which is difficult to achieve in the field, where realism often compromises control and replicability [4]. A key strength of VR is the active role of the user-learner, who interacts with the virtual environment and experiences accidents in a controlled, risk-free setting.

While adapting technology to the training scenario can be beneficial, it also presents challenges in assessing VR’s effectiveness in safety training. Renganayagalu et al. noted this difficulty when evaluating VR training integrated with workplace safety [8]. This issue will be explored in future research. Nonetheless, this study successfully identifies the demographic groups most influenced by the first VR course (SO1) in terms of their ability to recall the experience. 

This paper focuses on the development of a VR-based training course for occupational safety, specifically designed for workers and trainers in the open-pit quarrying industry. The study reports on the results of testing the first of four simulations that make up the overall training project. The aim is to assess the impact of this initial immersive VR experience on groups of users with varying characteristics and skill levels. Understanding the strengths and weaknesses of the first simulation will help inform the effectiveness of VR in the mining sector.

Section 2 outlines the materials and methods used in the project development and the first Specific Objective experiments. Section 3 presents the initial results of the headset experience, platform data, and Simula Solution analysis. The final section discusses the strengths and weaknesses of VR in the quarry sector and offers suggestions for future research. This study concludes with a summary of the results.

## 2. Materials and Methods

The project utilizes an innovative virtual reality (VR) training system that promises to enhance both the effectiveness and efficiency of the entire training process, from onboarding to progress tracking. The system is based on a dedicated platform that enables the creation of multiple training sessions while providing real-time monitoring of users’ progress and performance via a browser interface. This platform automates the steps for downloading and installing the VR application, allowing users to access courses simply by entering their personal identification number (PIN) on the VR training portal. Once activated by the training provider, the courses are made accessible to the users.

Each user’s session is tracked in real time, and the platform generates comprehensive aggregated reports at the end of the session, detailing all relevant data. The evaluation of the VR experiences follows an experimental methodology developed by Inail and the Laboratory of Virtual Reality at the University of Siena (LabVR UNISI). This methodology, which integrates questionnaires, platform data, and experimental sessions, is designed with a focus on standardization. 

The equipment used in the experiment consists of Meta Quest 2, all-in-one virtual and augmented reality headsets that can be managed in a network for teaching in minimal multi-user (dyad) sessions, paired with Oculus Touch controllers. These devices are provided by the Virtual Reality Laboratory of the University of Siena (LabVR UNISI). The laboratory is also equipped with advanced technologies such as the Cave Automatic Virtual Environment (CAVE), which is used to test the simulations created for the Oculus Quest 2. Moreover, the use of specialized software for the creation of digital content and personalized experiences on the Unity platform allows users to explore complex and interactive scenarios.

### 2.1. Study Design

The project is structured into three main phases.

Preliminary Analysis: This phase involves examining both quarry-related accidents, with a particular focus on the marble sector, as well as identifying the best practices and standards applicable to the industry.

Project Development: This phase focuses on identifying specific objectives (SOs) corresponding to the operational phases with the highest accident rates. It includes the development of VR courses targeting these critical phases, defining the content for the volunteer interview surveys (administered through FORMS), and configuring Simula Solution sessions for real-time tracking of user experiences.

**Figure 1:**
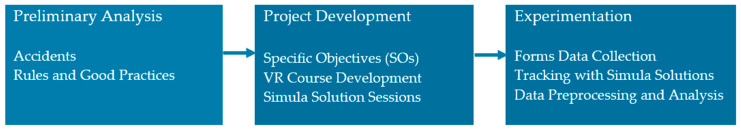
VR training project study design. Preliminary Analysis, Project Development, and Experimentation. Source: authors’ processing of accident data, mandatory rules, and good practices.

Experimentation: This phase encompasses the immersive VR simulations, the collection of data through FORMS and Simula Solution sessions, and the subsequent multivariate analysis of the results. An overview of these steps is summarized in Figure 1.

#### 2.1.1. Preliminary Analysis

This project is primarily inspired by the data from accident cases recorded in the quarry sector, available in the Inail archives (https://www.inail.it/portale/it/attivita-e-servizi/dati-e-statistiche/open-data.html, accessed on 12 November 2022). In addition to this source, data from the Accident Observatory of the Prevention Department (AOPD) of ASL USL Tuscany North-West, Department of Prevention—UOC Mining Engineering, were also included. These data, which cover accidents that occurred in the Apuan Alps marble quarries from 2006 to the end of 2022, are published in the contribution titled “Illustrazioni delle dinamiche infortunistiche in cava, dall’analisi alla prevenzione. Inail. Italy. 2023”.

Further content development was informed by Neural Network and Cluster Analysis techniques. These methods identified both qualitative and quantitative relationships between key terms (nodes) derived from the analysis of injury cases. The semantic representation of these nodes, based on their weight (diameter) and link strength (closeness), provided crucial insights into the most significant risk factors. Cluster analysis further refined the criteria for defining specific objectives (SOs) to be enacted in the virtual simulations of the project (Appendix A).

The resulting VR courses are content-driven, beginning with an in-depth analysis of raw materials through a systems approach. This approach identifies critical connections between process nodes and the structural causes of accidents. The content is grounded in the principles of the Functional Resonance Analysis Method (FRAM), which posits that the danger of certain activities or production phases increases with the variability in the system to which they belong. The training focuses on identifying these high-risk phases and developing simulations that help workers build skills to mitigate variability and introduce resilience into the system [11].

**Figure 2:**
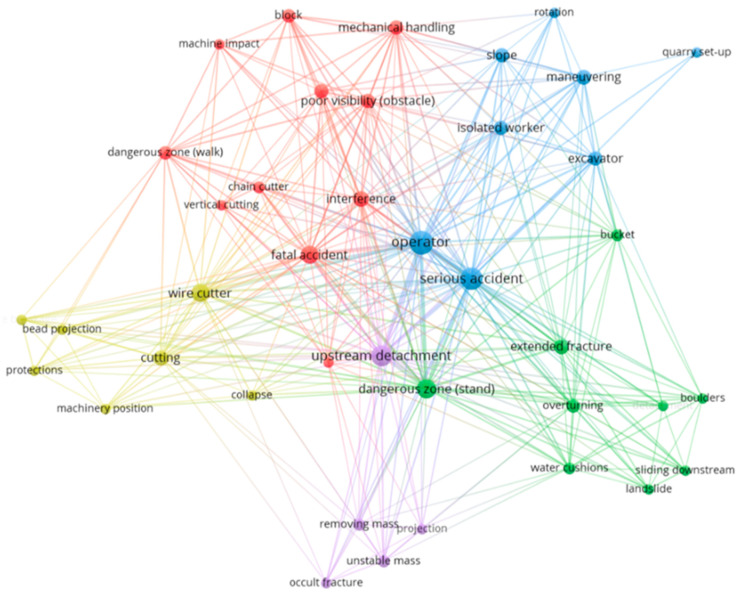
Preliminary analysis of serious and fatal accidents in open-cast quarries. Semantic representation of network analysis applied to serious and fatal accidents in marble quarries in Tuscany. Cluster 1 the red items, Cluster 2 the green items, Cluster 3 the blue items, Cluster 4 the yellow items and Cluster 5 the purple items. Source: authors’ processing on Inail and ASL USL Tuscany North-West AOPD data by R and Vos Viewer. Italy, years 2006–2022.

The preliminary analysis made it possible to determine the VR course content and thus the specific objectives (OSs) of the project (Table 1 and Figure 2). 

#### 2.1.2. Project Development

Each course encompasses a range of scenarios with varying contexts, based on methodologies (e.g., different cutting or tipping methods), risk situations (e.g., the presence of unstable masses, worn cutting tools, poorly configured cutting circuits, handling loads in confined spaces, or inadequate support conditions), and unforeseen events (e.g., the presence of operators in maneuvering areas). In each experience, the user navigates a quarry site composed of various areas separated by access slopes (scenes). For each scene, there is a distinct virtual environment (e.g., a yard or a quarry face), which may include machinery, tools, and virtual operators (characters that can interact in simple ways).

Starting from the lowest point, the user must explore the areas and apply the appropriate procedures in the varying contexts (e.g., selecting Personal Protective Equipment (PPE), choosing quarry methods, assessing risks, and responding to unforeseen events). The user will need to prepare, initiate, and supervise the different operations (functions) of the procedure. Within the virtual environment (e.g., near blocks, tools, or characters), the user can activate interactive hotspots and make decisions. To complete the course and achieve a high final assessment score, simply knowing the correct sequence of operations will not be enough. In addition to initiating the functions, the user must demonstrate an understanding of their implications. The goal is to complete and retain the VR experience.

#### 2.1.3. Experimentation

For each of the volunteers involved in the experiment, data were collected via Microsoft Forms and real-time monitoring (Simula solution software version 1.2.1) and then pre-processed for subsequent descriptive and multivariate analysis (MCA) in R.

The frequencies, correlations, and means of the data obtained from the test provided insights into the participants and their performance. Outlier detection and the Pearson correlation coefficient were calculated for each variable using R. 

Thus, the variables in the original dataset were reclassified to facilitate the interpretation of the results in the MCA dataset. MCA makes it possible to identify potential models and determine the most relevant variables for the interpretation of results. In addition, it makes it possible to reduce the dimensionality of the original variables by identifying synthetic explanatory variables (factors), which are linear combinations of the original categorical and/or ordinal variables, and to assess associations between the variables included in the model. This method was applied to test whether user characteristics were significantly correlated with the outcomes of the experience. Factors are extracted from orthogonal factorial axes, each explaining, in descending order, the maximum variability of the data matrix (inertia) [12,13,14]. 

## 3. Results

The network analysis and the information and elaborations shown in Table 1 and Figure 2 were taken from the Inail archives and from the archives of the Accident Observatory of the Prevention Department (AOPD) of Tuscany.

The network analysis in Figure 2 provides an overview of the semantic representation of the accident phenomenon, as described in the preliminary analysis. The details of each cluster and its associated items, including the most frequent ones along with their associative strength (TLS), are provided in Appendix A.

The eleven red items in Cluster 1 are block, chain cutter, communication, dangerous zone (walk), fatal accident, interference, machine impact, mechanical handling, pushing, vertical cutting, and poor visibility (obstacle). The nine green items in Cluster 2 are boulders, buckets, dangerous zone (stand), detachment, extended fracture, landslide, overturning, sliding downstream, and water cushions. The eight blue items in Cluster 3 include: excavator, isolated worker, maneuvering, operator, quarry setup, rotation, serious accident, and slope. Finally, the yellow and purple items, consisting of seven and five terms, respectively, are bead projection, collapse, cutting, machinery position, protections, wire breakage, wire cutter, occult fracture, projection, removing mass, unstable mass, and upstream detachment (Figure 2).

After determining the qualitative and quantitative parameters by means of the network analysis, it was possible to identify the specific objectives for the VR simulations to be prioritised. Accordingly, the “Course on primary upstream cutting” (SO1) was linked to the first cluster, the “Course on tipping banks” (SO2) was linked to the second cluster, the “Course on squaring and sectioning” (SO3) was linked to the fourth cluster and the “Course on handling and road transport” (SO4) was linked to the third and fifth clusters. A summary of the VR training courses to be included in the project are shown in Table 2.

### 3.1. The First VR Course on “Primary Upstream Cutting” (SO1)

The first course on Primary Upstream Cutting was implemented in an Italian marble quarry, at Siena University, and in the Inail research laboratory. The research team first identified potential users who met the necessary criteria to participate in the project. The inclusion criteria focused on competencies in Health and Safety (H&S) in workplaces, quarrying skills, and Virtual Reality (VR) skills, as well as the age of the users. The age criterion is often linked to the differing backgrounds of “tech natives,” who tend to have a more open approach to technological innovation and are generally more receptive to VR experiences. Finally, the inclusion criteria for participation in the study were (a) age between 20 and 60, and (b) at least two years of work or study experience. Based on these two key aspects, which were crucial for the outcome of this study, the research team identified 40 participants. The research team members explained the study objectives to the participants and collected written consent forms, along with completed questionnaires. In many cases, the team encountered resistance and skepticism regarding the VR training experiment, often due to previous experiences where participants had experienced symptoms of discomfort such as nausea, sweating, fear, and a sense of losing control.

**Figure 3:**
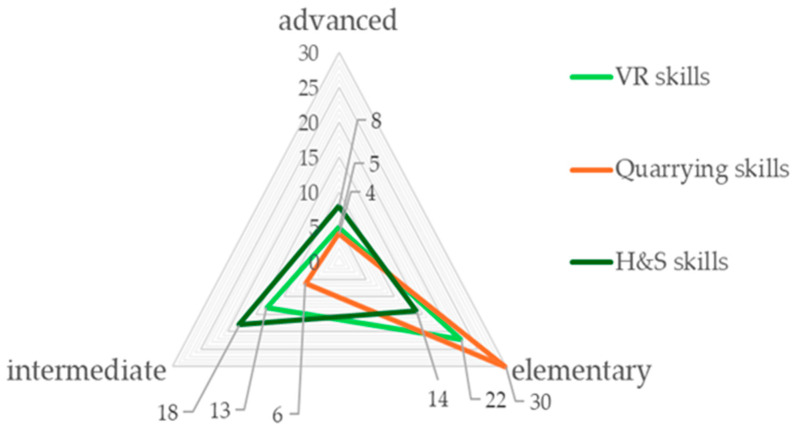
Skills in VR, mining and occupational health and safety of the 40 participants in the first course trial (SO1). Source: authors

The distribution of skills among the 40 volunteers was as follows: 55% had elementary VR skills, 33% intermediate skills and 12% advanced VR skills; 75% had elementary extraction skills, 15% intermediate skills and 10% advanced extraction skills; 35% had elementary H&S skills, 45% intermediate skills and 20% advanced H&S skills (Figure 3).

The resulting survey dataset included four quantitative variables (Table 3), ten qualitative variables (Table 4), and one text column containing user comments after the experience (Appendix B). The first 14 nominal categorical variables were used for multiple correspondence analysis (MCA) purposes. Five classes were defined for user characterization: user activity (quarryman and others, researcher, student); user age (20–35, 36–50, over 50); Health and Safety skills (advanced for specialists, intermediate for users with regular training, elementary for those with no training); mining skills (advanced for quarry workers, intermediate for technical experts, elementary for those with no knowledge of the sector); and virtual reality skills (advanced for regular VR users, intermediate for occasional users, elementary for first-time users).

For the experience characterization, five classes were defined: number of attempts (1–10), percentage of completion (up to 70% or 70–100%), score achieved (up to 70% or 70–100%), time needed to complete the experience (up to 10 min, 10–15 min, over 15 min), and user-reported discomfort (1–10). Retention of the experience was measured using two classes: Retention 1 (number of mistakes remembered by users, such as one error, all errors, no errors, or forgotten) and Retention 2 (type of error remembered: process, PPE, machinery, or forgotten). Opinions on the VR training course were collected before (Opinion 1) and one week after (Opinion 2) the experience, with scores ranging from 1 to 10.

**Figure 4:**
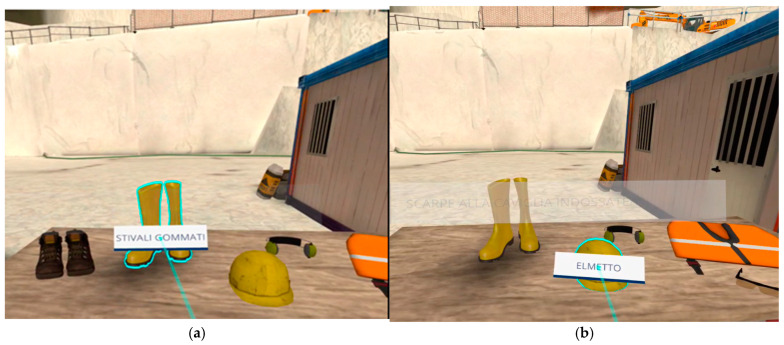
VR course on primary cutting upstream (SO1). The selection of Personal Protective Equipment (PPE) as the rubber boots (stivali gommati) or helmet (elmetto) is the first step upon entering the quarry site and precedes drilling and cutting activities using diamond wire cutting machines. The user interacts with the pointer to either evaluate (**a**) or wear (**b**) the most appropriate PPE. Source: authors’ elaboration using Software Simulation version 1.2.1.

All 40 participants were volunteers. The first course (SO1) was initially tested by the Inail and University of Siena research teams in early 2024, at the Franchi Umberto Marmi Company, which was involved in the project. The final experimentation with the 40 volunteers, along with the related measurements, was conducted at the Franchi Umberto Marmi Company, Siena University, and the Inail research laboratories. The VR course on primary upstream cutting (SO1) begins with the worker entering the quarry site, followed by the assessment and selection of the necessary PPE, as determined by the employer after conducting a Health and Safety (H&S) risk assessment (Figure 4).

**Figure 5:**
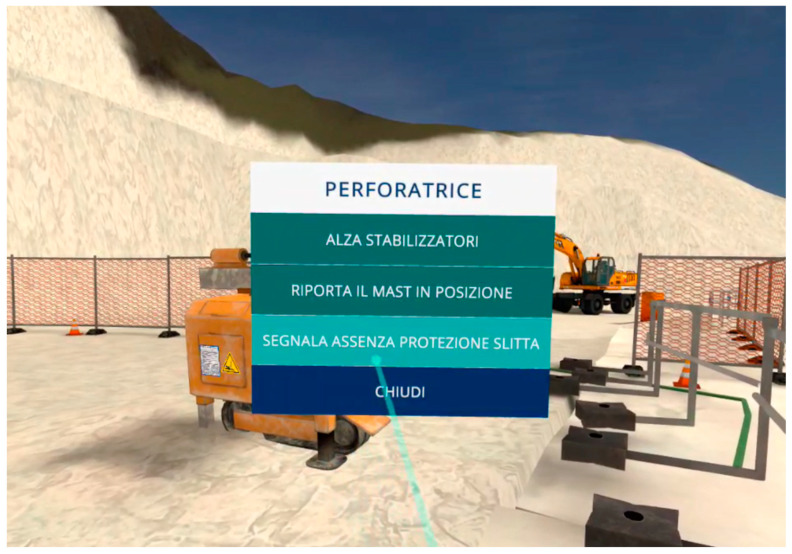
VR course on primary cutting upstream (SO1). Before starting, the user interacts with the pointer and verifies the compliance of the operational phase choosing from the distinct options. Source: authors’ processing by Software Simulation version 1.2.1.

**Figure 6:**
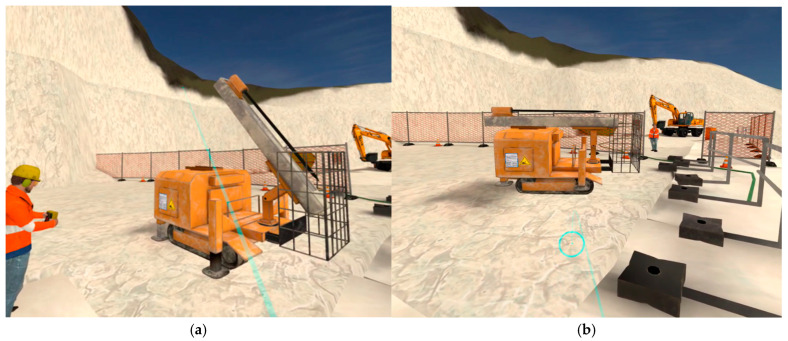
VR course on primary cutting upstream (SO1). In the drilling phase the user checks the worker, the site and machineries (**a**). A stranger worker in the drilling area represents a hazardous situation that the user must resolve (**b**). Source: authors’ processing by Software Simulation version 1.2.1.

All previous steps are subject to prior control by the user, who must make a series of choices from the options provided by the developer. Figure 5 illustrates an example of the available options, where the user controls the machine to complete the drilling process. The options for the drill (perforatrice) include i. raise the stabilizers; ii. return the slide to its position; and iii. arrange the slide guard. Once the cycle of checks has been completed, the user exits the menu (chiudi) and proceeds to the next steps (Figure 6). 

**Figure 7:**
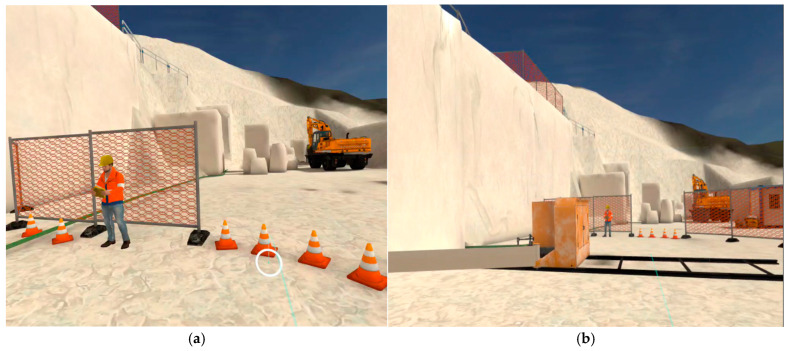
VR course on primary cutting upstream (SO1). Before starting horizontal cutting, the user interacts with the pointer, verifies the compliance of the operational phase checking the safety distances from the machine (**a**), positioning the diamond wire guards in order to avoid whiplash in the event of wire breakage during horizontal cutting (**b**). Source: authors’ processing by Software Simulation version 1.2.1.

**Figure 8:**
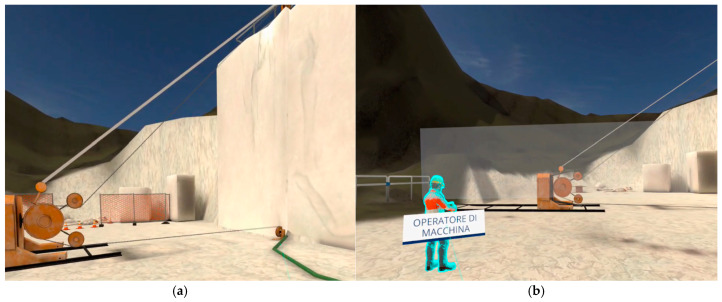
VR course on primary cutting upstream (SO1). Before starting vertical cutting, the user interacts with the pointer, verifies the compliance of the operational phase checking the safety distances from the machine (**a**), positioning the flexible and rear guards to prevent whiplash in the event of wire breakage during vertical cutting (**b**). Source: authors’ processing by Software Simulation version 1.2.1.

Once the drilling at height has been completed, the bank is set up for the insertion of the diamond wire. At this point, the operators move downstream to prepare and delimit the cutting area and position the cutting machine near the base of the marble bank to be detached by horizontal and vertical cutting (Figure 7 and Figure 8).

#### 3.1.1. Multiple Correspondence Analysis (MCA)

The MCA dataset consists of 40 individuals (volunteer users) and 14 variables, 4 of which are quantitative and considered illustrative (Table 3 and Table 4). The analysis of the graphs revealed no outliers. The factors to be considered in the MCA are determined based on their relevance, specifically the proportion of total inertia they explain. The number of factors was established graphically by identifying the inflection point on the total inertia graph. Beyond this point, adding another factor did not lead to a significant increase in the total variability. Using this approach, we identified three dimensions that accounted for over 45% of the total variance.

To interpret the most important factorial axes, we assessed which modalities contributed most to their determination. The characterization of each axis, according to the modalities, was based on the following aspects: the position of the modality on the factorial axis, its positive or negative sign (with 0 representing the mean of the coordinates), and the distance from 0. The further a modality’s coordinate value is from 0, the more it represents its overall significance. The absolute contribution expressed the importance of each modality in relation to the factor, i.e., how much a modality influenced the factor and helped interpret the axes. The relative contribution assessed how much a factor contributed to the reproduction of the dispersion (inertia) of each modality. This value ranges from 0 to 1, with higher values indicating that the factor better represents the modality.

#### 3.1.2. Description of Axes

**Figure 9:**
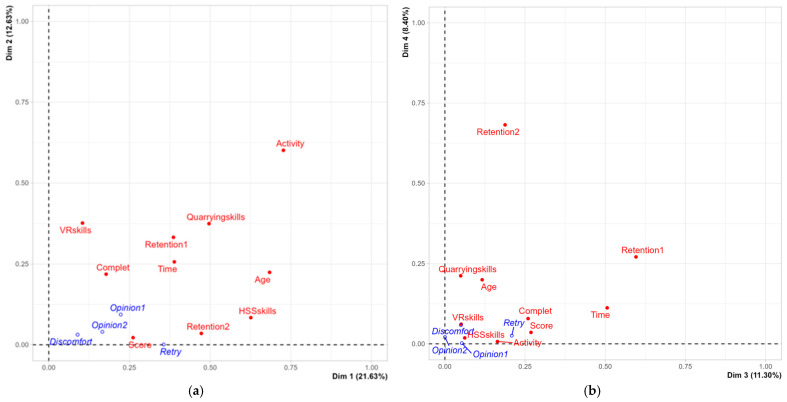
MCA. Variables factor map. The explanatory capacity of the variables in the plain Dim1Dim2 (**a**) and Dim3Dim4 (**b**) by the factorial axes. The qualitative variables are red while the quantitative ones are blue. Source: authors’ processing on MCA dataset by R.

For the purposes of representativeness in the construction of the axes (Figure 9), only the factors with R^2^ greater than or equal to 0.5 and *p*-value less than 0.05 are significant (Table 5).

**Figure 10:**
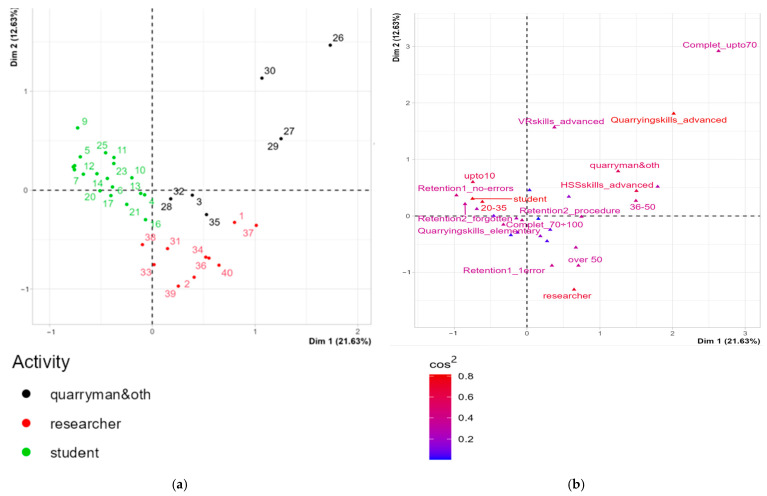
MCA. Individuals by user activity (**a**) and variables factor map by correlation (cos2) with axes (**b**) in the plain Dim1 and Dim2. The label provided higher contribution to the plane construction. Source: authors’ processing on MCA dataset by R.

As represented in Figure 10a, Dimension 1 opposes the group of users classified by activity (30, 27, 29, 26, 40, 39, 2, 34, 36, and 37) to the right on the graph, characterized by a strongly positive coordinate on Dim1 to a group of individuals (5, 7, 8, 12, 14, 17, 18, 25, 15, 19, and 9) on the left of the graph, characterized by a negative coordinate on Dim1. The most correlated variables (higher cosine) with the axes are in deeper red (Figure 10b).

Results of Dim1 (+) vs. Dim2 (+). The group of individuals 30, 27, 29, and 26 characterized by a positive coordinate on Dim1 and Dim2 (Figure 10a) shares high frequency to the factors Completion% = up to 70%; quarrying skills = advanced, activity = quarryman and other; Retention 1 = all errors; H&S skills = advanced; Retention 2 = procedure; age = 36–50 (Figure 10a,b).

Results of Dim1 (+) vs. Dim2 (−). The group of individuals 40, 39, 2, 34, 36, and 37, characterized by a positive coordinate on Dim1 and negative coordinate on Dim2 (Figure 10a), shares high frequency to the factors Retention 1 = 1 error; activity = researcher; quarrying skills = elementary; time min = 10 ÷ 15; age = over 50 (Figure 10a,b).

Results of Dim1 (−) vs. Dim2 (+). The group in which individuals 5, 7, 12, and 9 stand characterized by a negative coordinate on Dim1 and a positive coordinate on Dim2 (Figure 10a) shares high frequency for the factors age = 20–35; activity = student; time min = up to 10; Retention 1 = no errors; Retention 2 = forgotten; H&S skills = elementary; quarrying skills = elementary (Figure 10a,b).

**Figure 11:**
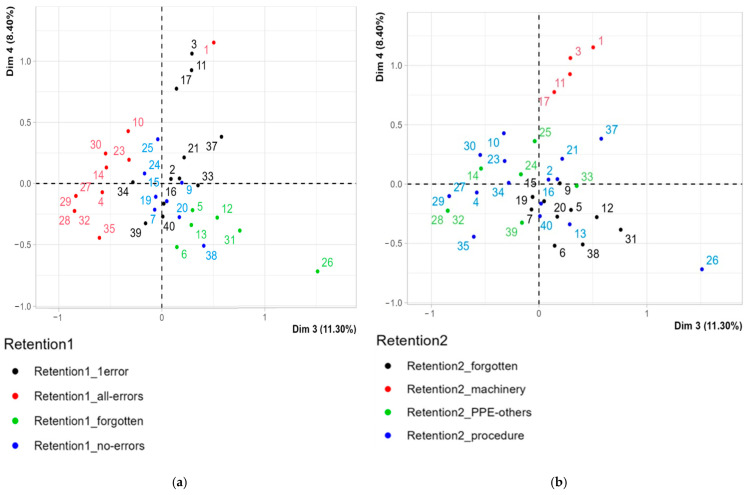
MCA. Individuals (users) by Retention 1 (**a**) and Retention 2 (**b**) in the plain Dim3 Dim4, according to Table 5. Source: authors’ processing on MCA dataset by R.

As represented in Figure 11, Dimension 3 opposes individuals such as 26, 31, 38, 12, and 6 to the right of the graph, characterized by a strongly positive coordinate on the axis, to individuals such as 28, 32, 35, 4, 27, 29, 14, and 10 to the left of the graph, characterized by a strongly negative coordinate on the axis (Figure 11a,b).

Results of Dim3 (+) vs. Dim4 (+). The group of individuals characterized by a positive coordinate on Dim3 and Dim4 shares high frequency to the factors Retention 2 = machinery; quarrying skills = intermediate (Figure 11a,b).

Results of Dim3 (+) vs. Dim4 (−). The group of individuals characterized by a positive coordinate on Dim3 and negative coordinate on Dim4, shares high frequency to the factors Retention 2 = forgotten, Retention 1 = forgotten; time min = over15; age = over 50.

Results of Dim3 (−). The group of individuals characterized by a negative coordinate on Dim3 shares high frequency for the factors Retention 1 = all errors, time min = 10 ÷ 15 and Retention 2 = procedure (Figure 11a,b).

**Figure 12:**
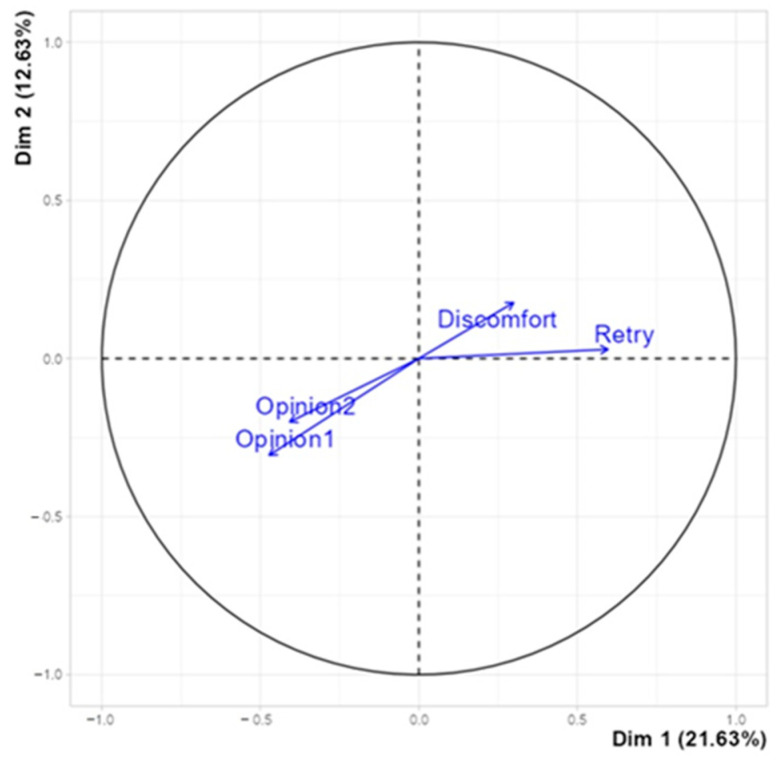
MCA. Quantitative factor map. The graph represents the inverse correlation between discomfort, retry (number of attempts before VR training completion), and high values of the opinions before and after the VR experience. Authors’ processing on MCA dataset by R.

Although MCA is primarily a multivariate analysis for qualitative variables, it is also useful for analyzing quantitative variables. In this context, the factor map illustrates the inverse correlation between high levels of physical discomfort, the number of attempts needed to complete the test, and the opinions expressed by participants regarding the virtual reality training. A summary of this relationship is shown in Figure 12, which corresponds to the first two dimensions.

## 4. Discussion

Safety training plays a crucial role in enhancing individuals’ ability to identify risks and assess their severity. These competencies are key determinants of human behavior and safety, particularly when performing hazardous tasks. Unsafe behaviors can lead to fatalities, injuries, and economic consequences, especially in the context of workplace accidents or disaster events. According to Sacks et al., safety training is a primary mechanism for improving human behavior in dangerous work environments [15]. In manufacturing operations, training can significantly benefit from the integration of virtual reality (VR) applications, which have proven effective in processes such as welding and other manufacturing tasks [16,17,18,19,20]. In the construction sector, Nykanen et al. explored the use of VR technology for safety training and concluded that it has the potential to improve safety competence and motivate behavioral changes. They also emphasized the need for further research on participatory human factors safety training, particularly through versions targeting both management and workers, delivered in extended formats [21].

Our study focuses on applying VR technology to the quarrying sector and its associated production processes, with an emphasis on safety. This choice is motivated by the severity of accidents in the sector (as documented by INAIL and ASL USL Tuscany North-West AOPD) and the resistance of this industry to adopting technological innovations, as identified in previous INAIL studies. These findings align with those of Scorgie et al., who noted that VR training has only a marginal impact in this sector due to its limited adoption. Although the concept of VR in safety training is not new, the use of immersive VR technology remains relatively uncommon [22].

Italy’s marble sector, with its thousand-year history, has left an indelible mark on sculptural works like Michelangelo’s Pietà and historical Italian architecture. This millennial tradition has preserved extraction methods passed down through generations, remaining largely unchanged. From our observations, this cultural heritage contributes to the sector’s resistance to adopting innovative, technology-driven methods, even those that offer enhanced safety. This resistance stems from a desire to protect the historical essence inherent in traditional extraction practices.

Administering VR training in this sector, while integrating safety principles with traditional extraction methodologies, could help address these resistances. By blending innovation with respect for historical practices, VR training has the potential to improve safety while preserving the cultural identity of quarrying operations.

Virtual reality (VR) technology enables users to visualize, manipulate, and interact with complex computer-generated data in an intuitive manner [23,24]. Essentially, VR immerses users in a digital environment, allowing them to engage with its components naturally through sensory inputs like vision, hearing, and touch. The key elements of VR applications are divided into two categories: sensors, which track user activity and provide input to the application, and visualization tools, which output data to the user [25,26]. Zhang evaluated the effects of each mining training system on 10 students and concluded that users are the most important factor that should receive more attention in VR mine training systems [27]. According to Karagiannisa et al., the primary benefits of VR tools lie in the safety of operating within a simulated environment combined with the intuitive engagement offered by immersive technology [28].

Retention measurements are particularly important when comparing the effectiveness of VR-based training to traditional methods. The disparity is most evident in long-term outcomes, as shown in studies reviewed by Chittaro and Buttussi and Lovreglio et al. [29,30]. These studies commonly measured retention over periods of 2 to 3 weeks, highlighting a significant limitation in assessing the long-term impact of safety training solutions. The importance of immediate feedback in VR training has been demonstrated by Feng et al. and Burigat and Chittaro. Their findings suggest that immediate feedback is an effective approach for delivering knowledge and enhancing self-efficacy in safety training contexts [31,32]. Scorgie et al. observed that only a small proportion (9.6%) of studies on VR explicitly incorporated theoretical frameworks during the development and testing of VR applications. They also highlighted a significant gap in long-term retention measurements, with only 36% of studies addressing this aspect [22]. Stefan et al. categorized the results of their review of virtual reality training according to the four levels of the Kirkpatrick model for training evaluation. The authors showed that most of the studies included in the review assessed levels of reaction (66.18%) and learning (72.06%), while very few studies assessed behavior and outcomes [9].

The theoretical framework for developing and testing VR safety training in the quarry industry is grounded in the findings outlined above. Case study SO1 represented the pilot course of the entire project. It was particularly challenging and full of critical issues starting from the implementation of the experience in the quarry to the final evaluation after the experience. It can be said that from a methodological point of view, it played a “pathfinder” role that it was right to exploit in this study. The remaining SO2, SO3 and SO4 experiences of the project constitute the further developments of the project. The strength of our experiment lies in the measurement criteria used to evaluate the performance of 40 participants. These criteria were based on a tracking system powered by Simula Solutions software version 1.2.1., which enabled us to capture all variables associated with each participant’s experience. An application of multivariate analysis (MCA) was used with the specific objective of testing whether user characteristics were significantly correlated with the outcome of the experience. This focused-on tracking using Simula Solutions software during the experience and analyzing responses provided through Forms by the volunteers after the experience. Outcomes were assessed using two key variables, Retention 1 and Retention 2 (Figure 11), which measure participants’ ability to recall information. Retention 1 gauges the number of errors made during the experience, while Retention 2 identifies the type of errors committed. The most significant factors influencing the results from the MCA were the participant’s activity, age, and skill levels.

The student group, primarily aged between 20 and 35, had low skills in H&S procedures and mining operations but moderate VR proficiency. This group showed a correlation with “Retention 1 = no errors”, indicating no “game out” events, and “Retention 2 = forgotten”, with completion times under 10 s. The “students” group, aged between 20 and 35, showed better performance in terms of time, completion percentage, and score achieved. Conversely, this same group performed poorly in terms of remembering both the number and type of mistakes made.

In contrast, the quarry workers and other employees, aged 36 to 50, exhibited high expertise in H&S and mining procedures, with moderate VR skills. Their performance was closely associated with “game out” events and “Retention 2 = procedures”, reflecting a correct sequential order of operational phases, with completion rates averaging about 70%. The “quarry workers” aged between 36 and 50 generally had worse performance in terms of time, percentage of completion, and score achieved but performed better in remembering both the number and type of errors made.

Finally, the group of researchers, mostly aged over 50, demonstrated medium to high expertise in H&S, elementary skills in mining procedures, and moderate VR proficiency. Their performance was characterized by completion rates ranging from 70% to 100%, with “Retention 1 = 1 error” (Figure 10).

A significant relationship was found between the activity carried out by the user (often linked to skills in H&S) and the ability to memorize, in the short term, (one week after the experience) the mistakes made that led to a reduction in the score achieved. The group dominated by students demonstrated excellent skills in managing the “serious game” but poor ability to memorize its contents and any errors after the experience. Previous Health and Safety and mining skills contributed positively to memorizing the experience and any virtual errors or injuries experienced by users. The MCA highlighted a correlation between the activity carried out by the user and the results obtained. The performances tracked during the experience were positively correlated with the student group, while the performances tracked after the experience, particularly in terms of the memorization of errors, were positively correlated with the “non-students” group (Figure 10).

These results lead us to conclude that the effectiveness of VR training does not solely rely on the performances tracked during the experience (e.g., time, score, and completion percentage) as seen in gaming but also on the ability to memorize the correct sequence of the simulation, the mistakes made, and the scenarios related to “virtual injuries”. The study demonstrates that the latter is influenced by the user’s prior knowledge and skills, emphasizing that VR training cannot disregard previous expertise on the subject.

Although these results are based on a small sample size, which limits their generalization, they align with the future direction identified by the legislator, who sees VR as an integrative tool for the current Health and Safety training courses in Italy. The legislator could thus enhance one of VR’s strengths by introducing a learner-centered approach, in contrast to the passive approach typically observed in Health and Safety training [33]. The immersive experience encourages the user to interact with the resources of the virtual work environment and experience virtual incidents in a controlled, risk-free environment, thus adding significant value to training for high-risk activities such as quarrying and construction.

Cultural resistance to innovative technologies like VR is still prevalent in these sectors, not only due to historical reasons but also because of the high variability in production cycles, which makes adapting the technology to the context particularly challenging. These aspects warrant further exploration in future studies. Beyond the developers’ efforts to improve the technological adaptation of VR training, the legislator can intervene by implementing experimental training plans and enhancing VR technology’s strengths, ultimately helping mitigate the limits and cultural barriers that characterize high-risk activities such as quarrying.

## 5. Conclusions

This study involved 40 participants and analyzed 15 variables, including occupation, age, H&S skills, process knowledge, equipment familiarity, VR skills, physical impact of VR, number of attempts before completion, percentage and time of completion, achieved scores, retention of experience, and user feedback before and after the training. The data revealed three distinct clusters:

- Participants aged 20–35 demonstrating low rates of “game over” events and reduced VR impact.
- Participants aged 36–50 with high VR impact and advanced mining process experience.
- Participants over 50 with gaming experience and moderate mining process familiarity.

The survey successfully identified which demographic groups are most influenced by VR training. The performances tracked during the experience were positively correlated with the group of students, while the performances tracked after the experience regarding the memorization of errors were positively correlated with the group of “non-students”. The most significant original variable was the type of activity performed by the user, followed by age. The characterization of “not completed” trials and the “number of attempts after the first” before completing the trial was particularly important for quarry workers and less so for young students. In terms of effectiveness, however, the key characteristic of the quarry workers’ group was their ability to remember the number and type of mistakes made during the VR training. This ability to recall errors was a crucial factor in the success of the VR training, indicating that previous knowledge and expertise play an important role in long-term retention and learning outcomes.

## Figures and Tables

**Table 1:** Preliminary analysis of serious and fatal injuries occurred in open pit quarry sector. Results of network analysis applied to serious and fatal accidents in marble Tuscany quarries. Source: authors’ processing on Inail and ASL USL Tuscany North-West AOPD data by R and VosViewer. Italy, years 2006–2022.

Network Parameters	Description
Subject:	30 serious and fatal quarry accidents
Period:	2006–2022
Type of data:	text
DM Method:	neural network analysis
Fmin Minimum Keyword (node) Occurrences:	2
Selected Nodes (items)/Total Nodes:	40/183
Arches (links):	343
Clusters:	5
Total Link Strength Association (TLS):	745
Radius of node:	weight based on occurrence
Closeness between nodes:	nodes association degree

**Table 2:** Scheduled project-specific objectives on VR training courses. Source: authors.

Specific Objectives (SOs)	Time
1. VR Course on primary upstream cutting (SO1)	6th month
2. VR Course on tipping banks (SO2)	12th month
3. VR Course on squaring and sectioning banks (SO3)	18th month
4. VR Course on bank handling and road transport (SO4)	24th month

**Table 3:** MCA dataset. Outcome on quantitative variables Opinion 1, Retry n. Opinion 2, on user performances and VR training experience on course OS1. Frequencies (Freq.) and percentages (Perc.). A total of 40 users. Source: authors’ processing on MCA dataset by R.

Opinion 1	Freq.	Perc.	Retry n.	Freq.	Perc.	Discomfort	Freq.	Perc.	Opinion 2	Freq.	Perc.
0	1	0.03	0	21	0.53	0	1	0.03	0	0	0.00
1	1	0.03	1	9	0.23	1	26	0.65	1	0	0.00
2	0	0.00	2	5	0.13	2	7	0.18	2	0	0.00
3	0	0.00	3	2	0.05	3	4	0.10	3	0	0.00
4	2	0.05	4	1	0.03	4	0	0	4	0	0.00
5	2	0.05	5	1	0.03	5	0	0	5	1	0.03
6	5	0.13	6	1	0.03	6	0	0	6	1	0.00
7	5	0.13	7	0	0.00	7	0	0	7	14	0.35
8	10	0.25	8	0	0.00	8	0	0	8	3	0.08
9	5	0.13	9	0	0.00	9	0	0	9	7	0.18
10	9	0.23	10	0	0.00	10	0	0	10	15	0.38

**Table 4:** MCA dataset. Outcome on qualitative variables on users, performances and opinion on Course OS1. Modes, frequencies and percentages. A total of 40 users. Source: authors’ processing on MCA dataset by R. * Personal Protective Equipment.

Title 1	Mode	Frequency	Percentage
Activity	quarryman and others	8	0.20
	researcher	10	0.25
	student	22	0.55
Age	20–35	25	0.63
	36–50	6	0.15
	over 50	9	0.23
H&S skills	advanced	8	0.20
	elementary	14	0.35
	intermediate	18	0.45
Quarrying skills	advanced	4	0.10
	elementary	30	0.75
	intermediate	6	0.15
VR skills	advanced	5	0.13
	elementary	22	0.55
	intermediate	13	0.33
Completion%	70 ÷ 100	39	0.98
	up to 70	1	0.03
Score pt	up to 70	3	0.08
	70 ÷ 100	37	0.93
Time min	10 ÷ 15	12	0.30
	over 15	12	0.30
	up to 10	16	0.40
Retention 1	1 error	12	0.30
	all errors	11	0.28
	no errors	11	0.28
	forgotten	6	0.16
Retention 2	process	16	0.40
	PPE * other	7	0.19
	machinery	4	0.10
	forgotten	13	0.33

**Table 5:** MCA. Description of axes. The table expresses the explanatory capacity of the variables in the composition of the factorial axes for each individual dimension. Factors that are significant for the study are associated with R^2^ ≥ 0.5 and *p*-value < 0.05. Source: authors’ processing on MCA dataset by R.

Dim1			Dim2			Dim3		
Factor	R^2^	*p*-Value	Factor	R^2^	*p*-Value	Factor	R^2^	*p*-Value
Activity	0.73	3.69 × 10^−11^	Activity	0.60	4.16 × 10^−5^	Retention 1	0.60	3.16 × 10^−7^
Age	0.70	5.47 × 10^−10^	VR skills	0.38	1.59 × 10^−1^	Time min	0.50	2.15 × 10^−6^
H&S skills	0.63	1.25 × 10^−8^	Quarrying skills	0.37	1.68 × 10^−1^	Score pt	0.27	6.15 × 10^−4^
Quarrying skills	0.50	3.07 × 10^−6^	Retention 1	0.33	2.04 × 10^0^	Completion%	0.26	7.89 × 10^−4^
Retention 2	0.50	3.43 × 10^−5^	Completion%	0.22	2.36 × 10^0^	Activity	0.16	3.65 × 10^−2^
Time min	0.40	1.10 × 10^−4^	Time min	0.30	4.14 × 10^0^			
Retention 1	0.40	4.81 × 10^−4^	Age	0.22	9.13 × 10^0^			
Score pt	0.30	7.50 × 10^−4^						
Completion%	0.20	6.74 × 10^−3^						

**Figure A1:**
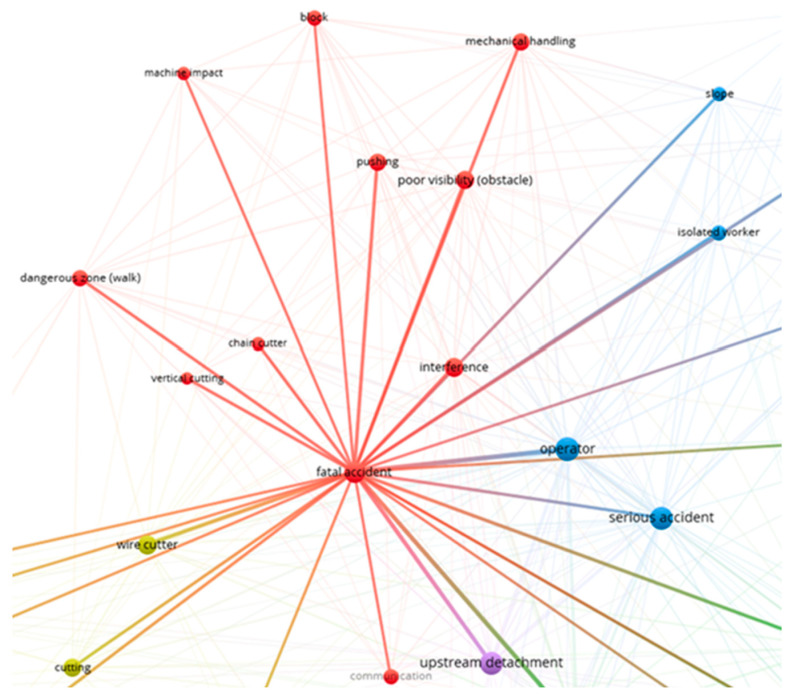
The eleven red items in Cluster 1: block, chain cutter, communication, dangerous zone (walk), fatal accident, interference, machine impact, mechanical handling, pushing, vertical cutting, and poor visibility (obstacle). SO1. Focus on fatal accident: 30 links, 72 as TLS. OS1.

**Figure A2:**
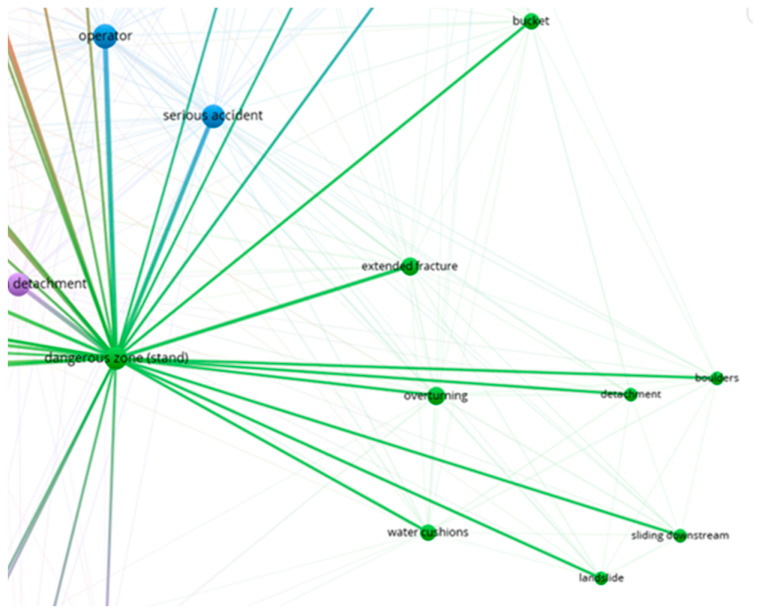
The nine green items in Cluster 2: boulders, buckets, dangerous zone (stand), detach-ment, extended fracture, landslide, overturning, sliding downstream, and water cushions (in purple “upstream detachment”). Focus on dangerous zone (stand): 33 links, 92 as TLS. OS2.

**Figure A3:**
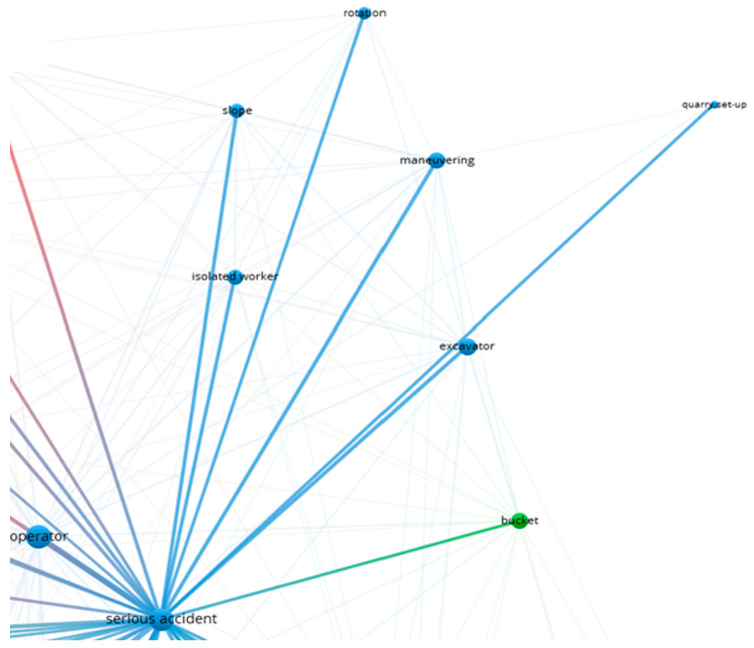
The eight blue items in Cluster 3: excavator, isolated worker, maneuvering, operator, quarry setup, rotation, serious accident, and slope. Focus on Serious accident: 38 links, 126 as TLS. OS4.

**Figure A4:**
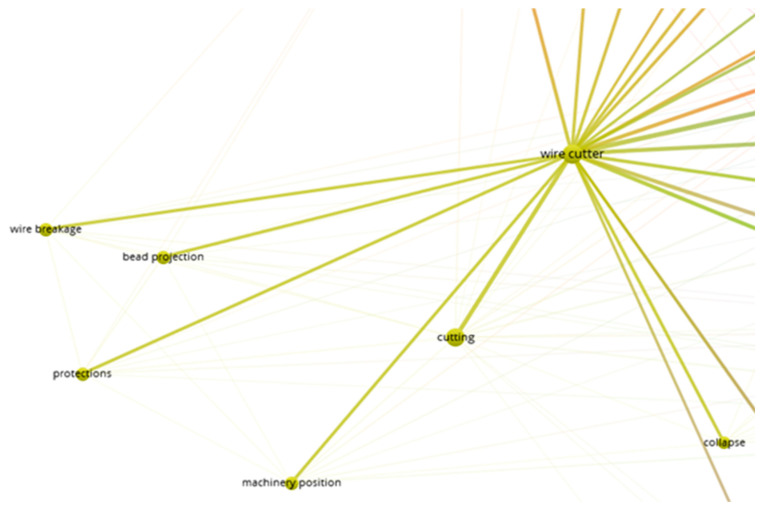
The seven yellow items in Cluster 4: bead projection, collapse, cutting, machinery position, protections, wire breakage, wire cutter. Focus on wire cutter: 24 links 70 as TLS. OS3.

**Figure A5:**
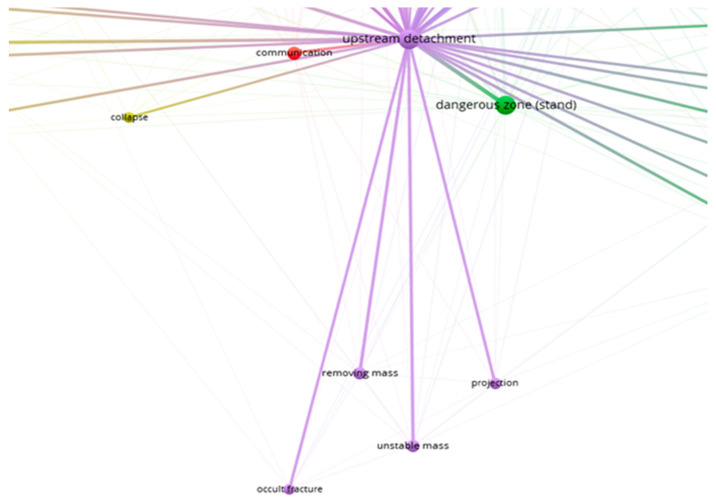
The five purple items in Cluster 5: occult fracture, projection, removing mass, unstable mass, and upstream detachment. Focus on upstream detachment: 37 links, 117 as TLS 117. OS4.

## Data Availability

Data is unavailable due to privacy or ethical restrictions.

## Appendix B

**Table A1:** User comments after the OS1 VR training experience.

Id	Comment
1	ND *
2	ND
3	nice experience
4	ND
5	ND
6	good
7	very interesting and formative
8	interesting and immersive
9	useful
10	very interesting
11	interesting
12	useful
13	ambiguous
14	new and positive
15	very realistic
16	beautiful
17	good
18	very interesting
19	nice experience useful for work
20	ND
21	useful for train VR
22	the guy with the headphones…
23	explanatory and educational
24	instructive, interactive and useful
25	useful and very stimulating
26	ND
27	ND
28	ND
29	ND
30	ND
31	ND
32	good
33	none
34	ND
35	good
36	interesting experience. Not having experience in quarry sector but only in H&S sector in general, the use of the system is still intuitive
37	interesting
38	ND
39	ND
40	no

* No comment available.
